# Impact of Image Interpretation of Carotid Ultrasound Findings on Lipid Management in Patients With Elevated ASCVD Risk

**DOI:** 10.1002/brb3.70956

**Published:** 2025-11-26

**Authors:** Xiaochuan Liu, Sichen Yao, Hua Yang, Zhigang Pan

**Affiliations:** ^1^ Department of General Practice Zhongshan Hospital, Fudan University Shanghai China; ^2^ Department of General Practice Wujing Community Health Service Center Shanghai China

**Keywords:** carotid ultrasound, general practitioners, image interpretation, low‐density lipoprotein

## Abstract

**Background:**

This study aimed to explore the impact of carotid ultrasound finding interpretation by general practitioners (GPs) on patients’ lipid management.

**Methods:**

This study is an observational cohort study involving elderly patients aged ≥ 60 years with elevated risk of atherosclerotic cardiovascular disease (ASCVD). Participants were divided into two groups: image interpretation group and routine care group, and then followed for 3 months. Our primary outcome was to explore the between‐group differences in low‐density lipoprotein (LDL) changes between the two groups at baseline and after 3 months of follow‐up. Secondary outcomes included between‐group differences in changes of total cholesterol (TC), triglyceride (TG), and high‐density lipoprotein (HDL). The Generalized Estimating Equation model was used to calculate interaction *p* values, *β* coefficients, and their 95% CIs.

**Results:**

This study enrolled 338 participants, with 169 in the image interpretation group (age 69.6 ± 5.76 years) and 169 in the routine care group (age 71.59 ± 5.26 years). The between‐group difference in LDL change from baseline to follow‐up was statistically significant (interaction *p = *0.028), with a *β* coefficient of −0.38 (95% CI −0.71, −0.04), indicating that the image interpretation group had a 0.38 mmol/L greater LDL reduction than the routine care group. This remained significant after adjusting for confounders (interaction *p = *0.025, *β* = −0.38, 95% CI −0.70, −0.05). There were no significant between‐group differences observed against changes in TC, HDL, and TG.

**Conclusion:**

This study indicated that adding image interpretation of carotid ultrasound results to routine care by GPs significantly improves lipid management in elderly patients with elevated ASCVD risk.

## Graphical abstract

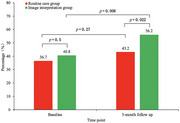

## 1 Introduction

Atherosclerotic cardiovascular disease (ASCVD) is a leading cause of mortality and disability in China (Liu et al. 2019). Data show that ASCVD accounted for approximately 25% of all deaths in China in 2016 (Zhao et al. 2019). With the accelerating aging of the population, the disease burden of ASCVD will become more severe in the future.

In recent years, multiple large clinical studies have demonstrated that early detection of subclinical atherosclerotic lesions via coronary computed tomography angiography (CTA) or carotid ultrasound, combined with interpretation of imaging results during physician–patient communication, can reduce the risk of ASCVD, improve lipid‐lowering medication adherence, and enhance lipid management (Diederichsen et al. 2015; Lindholt et al. 2022; Orakzai et al. 2008; Näslund et al. 2019). Similar strategies applied to smoking cessation education have shown significantly improved patient initiative to quit smoking (Shahab et al. 2007; Rodondi et al. 2008). Korcarz et al. (2008) found that handheld ultrasound screening for carotid atherosclerosis during patient visits improved cardiovascular literacy, with more patients expressing intentions to take lipid‐lowering drugs and clinicians adopting more guideline‐concordant treatment strategies. Notably, these studies were conducted by foreign researchers, and no domestic studies have explored whether interpreting subclinical atherosclerotic imaging can improve ASCVD risk factor management.

Dyslipidemia, as a critical and modifiable risk factor for ASCVD, is a common chronic disease managed by general practitioners (GPs) in primary care. Our previous study showed that GPs, after training, can use intelligent point‐of‐care ultrasound (POCUS) to screen for carotid plaque and interpret imaging findings with patients (Sun et al. 2025). Whether this intervention positively impacts lipid management remains unevaluated. Therefore, this study aims to investigate the effect of GPs interpreting carotid ultrasound findings on patients’ lipid management.

## 2 Methods

### 2.1 Study Design and Population

This study is an observational cohort study involving elderly patients aged ≥ 60 years with an elevated risk of ASCVD who seek medical help from primary community health centers. Participants were divided into two groups: (1) image interpretation group: patients underwent carotid plaque screening using intelligent POCUS by GPs, who then provided visual explanations of findings based on images. Concomitant health education on ASCVD and comprehensive lipid management were administered. Details of the screening protocol and criteria of enrolled participants can be found in Supporting Information. (2) Routine care group: patients received standard lipid management guidance by GPs, including ASCVD risk assessment, health education, pharmacotherapy, and lifestyle recommendations, without ultrasound‐based image interpretation. Participants in this group were matched to the image interpretation group by gender and age (± 5 years); no additional matching was performed for baseline lipid parameters or other clinical characteristics.

Data collection included medical history of chronic diseases (hypertension, diabetes, and dyslipidemia), obesity, smoking history, lipid‐lowering medication use, blood pressure, fasting blood glucose, and fasting lipid profiles (using levels within 2 weeks prior to enrollment as baseline, with supplementary lipid tests for missing records). Lipid‐lowering medications encompass three main classes (statins, ezetimibe, PCSK9 inhibitors, and bempedoic acid) of drugs, consistent with the lipid management guideline. Post‐enrollment lipid‐lowering medication use was recorded, and patients were invited for follow‐up visits 3 months (± 7 days) later to re‐evaluate medication use and lipid parameters. Data for the routine care group were collected via retrospective review of outpatient and lipid test records.

This study was approved by the Ethical Committee of Zhongshan Hospital, Fudan University. Informed consent was obtained from all participants prior to their enrollment. The reporting of this study adheres to the guidelines for observational research (von Elm et al. 2007).

### 2.2 Definition of Outcomes

Our primary outcome was to explore the between‐group differences in low‐density lipoprotein (LDL) changes between the image interpretation group and the routine care group at baseline and after 3 months of follow‐up. Secondary outcomes included between‐group differences in changes of total cholesterol (TC), triglyceride (TG), and high‐density lipoprotein (HDL). Additionally, we explored changes in lipid‐lowering medication use at baseline, post‐enrollment, and after 3 months of follow‐up in both groups, as well as changes in LDL target attainment rate (LDL < 2.6 mmol/L for patients without diabetes and LDL < 1.8 mmol/L for those with diabetes [Li et al. 2023]) at baseline and after 3 months of follow‐up. Furthermore, the impact of positive findings (carotid plaque detected) on outcomes in the image interpretation group was also explored. The definition of carotid plaque can be found in the Supporting Information.

### 2.3 Statistical Analysis

This study used STATA 15.0 for statistical analysis, with a significance level set at *p < *0.05. Continuous variables were tested for normal distribution using the Skewness and Kurtosis test. Normally distributed data were expressed as mean ± standard deviation (SD), and group comparisons were performed using the *t*‐test; otherwise, data were presented as median (interquartile range, IQR), and group comparisons used the rank‐sum test. Categorical variables were described as frequency (percentage), with group comparisons using the chi‐square test or Fisher's exact test; Bonferroni adjustment was applied for multi‐group comparisons. Given the potential correlation between baseline and 3‐month follow‐up lipid profiles, and the non‐normal distribution of lipid indices (LDL, TC, TG, HDL) confirmed by tests, a Generalized Estimating Equation (GEE) model was used to analyze between‐group differences in index changes over time. The model included an interaction term for group (routine care/imaging interpretation) and time (baseline/3‐month follow‐up), with calculation of interaction *p*‐values, *β* coefficients, and their 95% confidence intervals (CIs). Covariates with baseline group comparison *p < *0.1 were included to reduce confounding. Seven predefined subgroups were analyzed for heterogeneity in the effect of image interpretation versus routine care: gender (male/female), smoking status (former smoker/never smoker/current smoker), hypertension (yes/no), diabetes (yes/no), dyslipidemia (yes/no), baseline lipid‐lowering medication use (yes/no), and obesity (yes/no). Sample size consideration and sensitivity analysis are shown in the Supporting Information.

## 3 Results

### 3.1 Study Flowchart

**FIGURE 1:**
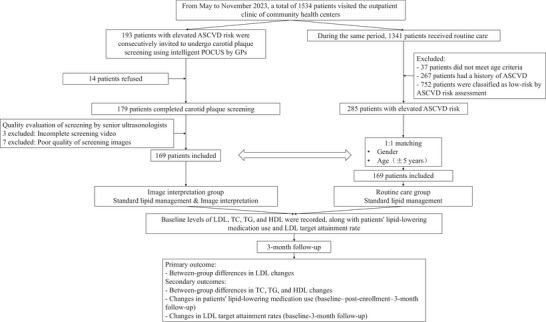
Study flowchart. ASCVD: atherosclerotic cardiovascular disease; GP: general practitioner; HDL: high‐density lipoprotein; LDL: low‐density lipoprotein; TC: total cholesterol; TG: triglyceride.

Figure 1 illustrates the study flowchart. From May to November 2023, a total of 1534 patients visited the outpatient clinic of community health centers. Among them, 193 elderly patients with elevated ASCVD risk were consecutively invited to undergo carotid plaque screening using intelligent POCUS by GPs, followed by image interpretation. A total of 14 patients refused participation, and some were excluded due to poor image quality unsuitable for evaluation, leaving 169 patients for final analysis in the image interpretation group. During the same period, 1341 patients received routine care; after excluding those ineligible, 285 patients were selected for 1:1 matching with the image interpretation group. Eventually, 169 patients were successfully matched as the routine care group. Operators were blinded to characteristics other than those required for matching. Both groups completed the 3‐month follow‐up.

### 3.2 Baseline Characteristics of Study Participants

This study enrolled 338 participants, with 169 in the image interpretation group and 169 in the routine care group. Baseline characteristics of the two groups are shown in Table 1. The routine care group had a mean age of 71.59 (± 5.26) years, significantly higher than 69.6 (± 5.76) years in the image interpretation group (*p < *0.001). A higher proportion of patients in the routine care group had hypertension (78.7% vs. 66.3%, *p = *0.015), while the image interpretation group had a higher proportion of patients already taking lipid‐lowering medication at baseline (21.3% vs. 12.4%, *p = *0.041). In terms of lipid indices, except for lower TG in the image interpretation group (median 2.5 [1.11, 3.46] mmol/L vs. 2.94 [2.04, 3.67] mmol/L, *p < *0.001), no significant differences were observed between the groups for other indicators (all *p > *0.05). The proportion of patients with diabetes was higher in the routine care group, but the difference did not reach statistical significance (65.1% vs. 55.0%, *p = *0.075).

**TABLE 1:** Baseline characteristics of study participants.

	Routine care group	Image interpretation group	
*N*	169	169	*p* value
Age	71.59 (5.26)	69.6 (5.76)	< 0.001
Gender			1.0
Male	64 (37.9%)	64 (37.9%)	
Female	105 (62.1%)	105 (62.1%)	
BMI	24.91 (22.86, 26.67)	24.97 (22.83, 27.14)	0.81
SBP, mmHg	130 (120, 132)	130 (122, 132)	0.68
DBP, mmHg	80 (78, 80)	80 (74, 82)	0.27
Smoking history			0.77
Never smoked	131 (77.5%)	135 (79.9%)	
Previously smoked, currently quit	21 (12.4%)	21 (12.4%)	
Currently smoking	17 (10.1%)	13 (7.7%)	
Risk for ASCVD			0.62
Moderate risk	1 (0.6%)	3 (1.8%)	
High risk	168 (99.4%)	166 (98.2%)	
Hypertension			0.015
No	36 (21.3%)	57 (33.7%)	
Yes	133 (78.7%)	112 (66.3%)	
Diabetes			0.075
No	59 (34.9%)	76 (45.0%)	
Yes	110 (65.1%)	93 (55.0%)	
Dyslipidemia			0.91
No	63 (37.3%)	61 (36.1%)	
Yes	106 (62.7%)	108 (63.9%)	
Lipid‐lowering medication			0.041
No	148 (87.6%)	133 (78.7%)	
Yes	21 (12.4%)	36 (21.3%)	
LDL target attainment rate			0.5
No	107 (63.3%)	100 (59.2%)	
Yes	62 (36.7%)	69 (40.8%)	
Blood test			
Glucose, mmol/L	7.65 (6.37, 9.12)	7.34 (6.35, 8.81)	0.53
LDL, mmol/L	2.93 (2.1, 3.1)	2.93 (2.09, 3.45)	0.57
TC, mmol/L	4.79 (3.7, 5.74)	4.79 (4.03, 5.66)	0.94
TG, mmol/L	2.94 (2.04, 3.67)	2.5 (1.11, 3.46)	< 0.001
HDL, mmol/L	1.28 (.93, 1.62)	1.28 (.93, 1.62)	0.93

*Note*: Data are presented as mean ± standard deviation, median (interquartile range), or count (percentage). Risk for ASCVD was assessed referring to Risk Assessment Flowchart.

Abbreviations: BMI: body mass index; HDL: high‐density lipoprotein; LDL: low‐density lipoprotein; TC: total cholesterol; TG: triglyceride.

### 3.3 Impact of Image Interpretation on Outcomes

Table 2 presents the primary and secondary outcomes. At baseline, there was no significant difference in LDL levels between the two groups (*p = *0.57). At 3‐month follow‐up, the image interpretation group had lower LDL levels (median 1.74 [1.18, 3.15] mmol/L vs. 2.9 [1.39, 3.76] mmol/L, *p < *0.001). The between‐group difference in LDL change from baseline to follow‐up was statistically significant (interaction *p = *0.028), with a *β* coefficient of −0.38 (95% CI −0.71, −0.04), indicating that the image interpretation group had a 0.38 mmol/L greater LDL reduction than the routine care group. This remained significant after adjusting for confounders (interaction *p = *0.025, *β* = −0.38, 95% CI −0.70, −0.05). For TC, no significant between‐group differences were observed at either time point, and the change from baseline to follow‐up did not differ significantly between groups (interaction *p = *0.515, *β* = 0.12, 95% CI −0.25, 0.50), remaining non‐significant after confounding adjustment (interaction *p = *0.508, *β* = 0.12, 95% CI −0.24, 0.49). TG levels were significantly higher in the routine care group at both baseline (median 2.94 [2.04, 3.67] mmol/L vs. 2.5 [1.11, 3.46] mmol/L, *p < *0.001) and follow‐up (median 2.08 [1.73, 2.33] mmol/L vs. 1.76 [1.46, 1.97] mmol/L, *p < *0.001). However, the between‐group difference in TG change from baseline to follow‐up was not significant (interaction *p = *0.964, *β* = −0.02, 95% CI −0.71, 0.68), remaining non‐significant after adjustment (interaction *p = *0.964, *β* = −0.02, 95% CI −0.71, 0.68). HDL levels did not differ significantly between groups at either time point (*p > *0.05), and the between‐group difference in HDL change from baseline to follow‐up was not significant (interaction *p = *0.686, *β* = 0.05, 95% CI −0.2, 0.3), remaining non‐significant after adjustment (interaction *p = *0.685, *β* = 0.05, 95% CI −0.19, 0.3). In image interpretation group, patients with carotid plaque showed significantly greater reductions in LDL and TC, and increases in HDL at 3‐month follow‐up compared to those without plaque. Image interpretation was more effective in reducing LDL among patients with hypertension, obesity, those never‐smoked, and those non‐lipid‐lowering medication users at baseline. Sensitivity analyses are consistent with our main results (Tables S1–S3).

**TABLE 2:** Impact of image interpretation on lipid management.

		Routine care group	Image interpretation group		Model 1			Model 2		
		169	169	*p*	interaction *p*	*β*	95% CI	interaction *p*	*β*	95% CI
Primary outcome										
LDL	Baseline	2.93 (2.1, 3.1)	2.93 (2.09, 3.45)	0.57	0.028	−0.38	(−0.71, −0.04)	0.025	−0.38	(−0.7,−0.05)
	3‐Month follow‐up	2.9 (1.39, 3.76)	1.74 (1.18, 3.15)	< 0.001						
Secondary outcomes										
TC	Baseline	4.79 (3.7, 5.74)	4.79 (4.03, 5.66)	0.94	0.515	0.12	(−0.25, 0.5)	0.508	0.12	(−0.24, 0.49)
	3‐Month follow‐up	3.73 (2.86, 4.58)	4.0 (2.79, 4.96)	0.36						
TG	Baseline	2.94 (2.04, 3.67)	2.5 (1.11, 3.46)	< 0.001	0.964	−0.02	(−0.71, 0.68)	0.964	−0.02	(−0.71, 0.68)
	3‐Month follow‐up	2.08 (1.73, 2.33)	1.76 (1.46, 1.97)	< 0.001						
HDL	Baseline	1.28 (0.93, 1.62)	1.28 (0.93, 1.62)	0.93	0.686	0.05	(−0.2, 0.3)	0.685	0.05	(−0.19, 0.3)
	3‐Month follow‐up	1.41 (1.08, 1.95)	1.37 (1.07, 1.91)	0.76						

*Note*: The *p* value represents the statistic for between‐group comparison of each index at two time points. The interaction *p* value is the statistic for the interaction term constructed by group (routine care group/image interpretation group) and time (baseline/3‐month follow‐up). Model 1 is the original model, and Model 2 is adjusted for confounders, including age, hypertension, diabetes, and baseline lipid‐lowering medication use. Lipid indices are in mmol/L.

Abbreviations: CI: confidence interval; HDL: high‐density lipoprotein; LDL: low‐density lipoprotein; TC: total cholesterol; TG: triglyceride.

### 3.4 Between‐Group Changes in LDL Target Attainment Rate

**FIGURE 2:**
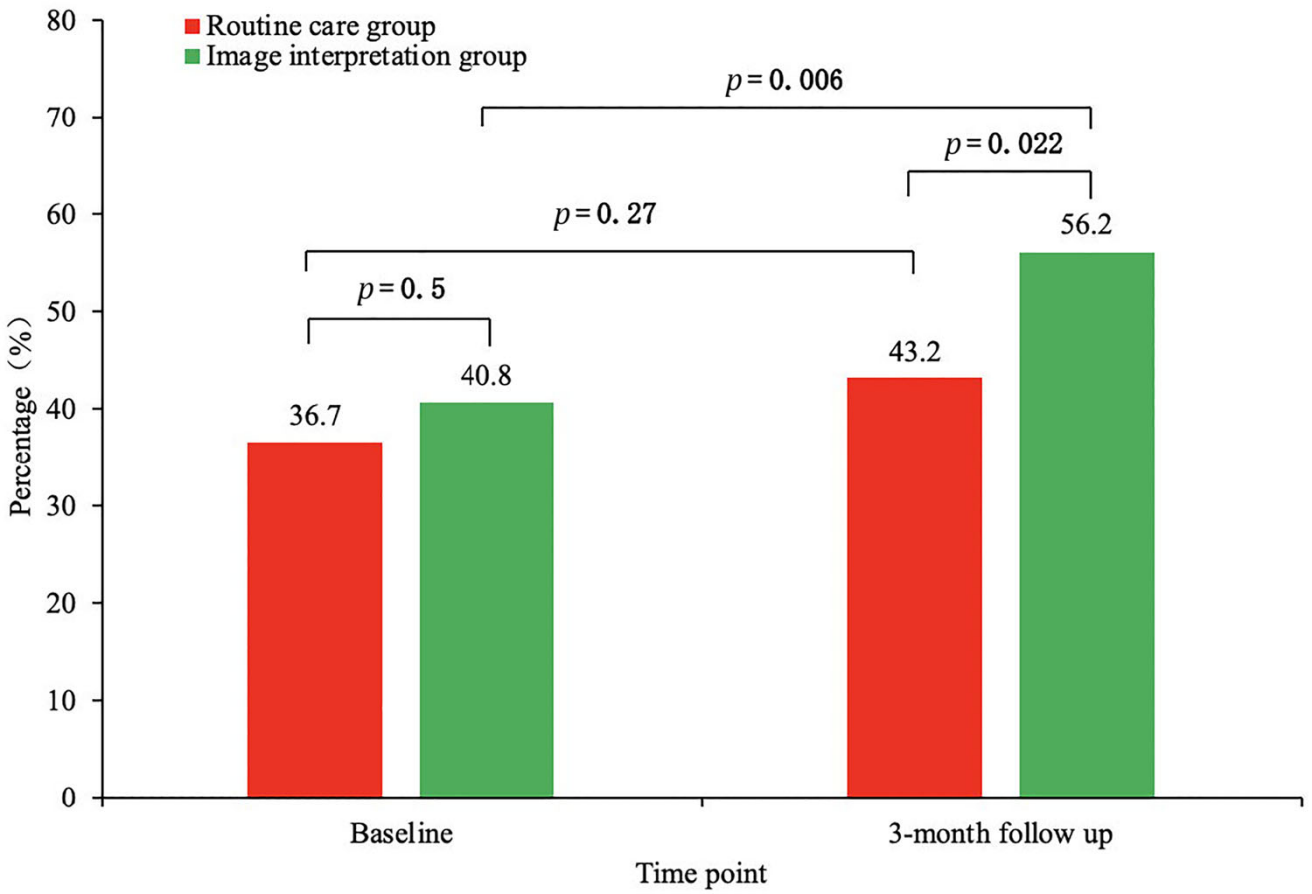
Between‐group changes in LDL target attainment rate.

At baseline, the LDL target attainment rate was 40.8% in the image interpretation group and 36.7% in the routine care group, with no significant difference (*p = *0.5). After 3 months of follow‐up, the LDL target attainment rate was 56.2% in the image interpretation group and 43.2% in the routine care group, showing a significant difference (*p = *0.022). The LDL target attainment rate in the routine care group increased from 36.7% to 43.2%, which was not statistically significant (*p = *0.27), while that in the image interpretation group increased from 40.8% to 56.2%, showing a statistically significant increase (*p = *0.006), as shown in Figure 2. LDL target attainment rates also rose markedly in patients with carotid plaque (Figure S1).

### 3.5 Between‐Group Changes in the Use of Lipid‐Lowering Medication

**FIGURE 3:**
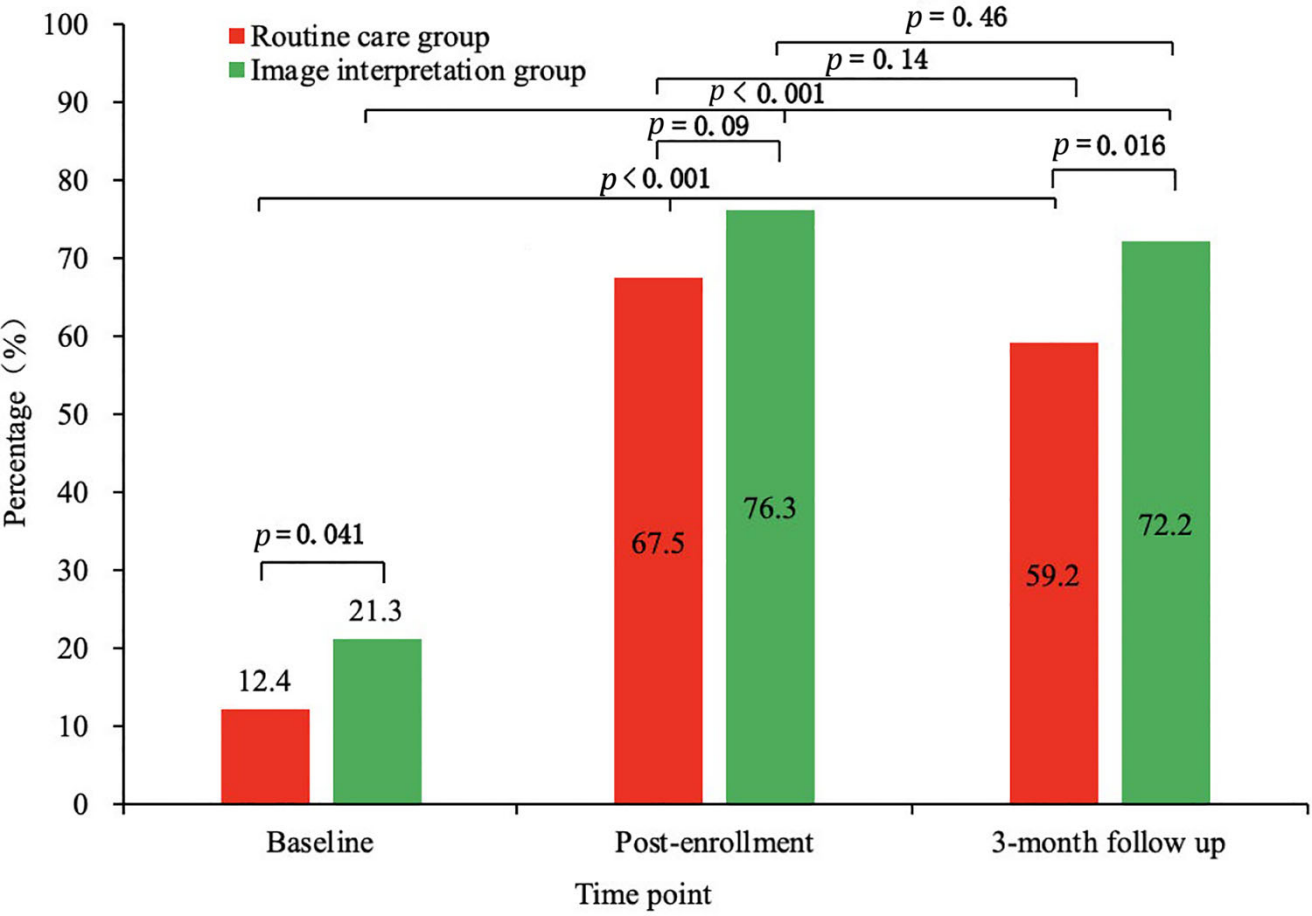
Between‐group changes in lipid‐lowering medication use.

We conducted cross‐sectional and longitudinal comparisons of lipid‐lowering medication use between groups at baseline, post‐enrollment, and 3‐month follow‐up (Figure 3). At baseline, the image interpretation group had a higher proportion of lipid‐lowering medication use than the routine care group (21.3% vs. 12.4%, *p = *0.041). There was no significant between‐group difference in the proportion of lipid‐lowering medication use post‐enrollment (76.3% vs. 67.5%, *p = *0.09), but the image interpretation group showed a significantly higher proportion at 3‐month follow‐up (72.2% vs. 59.2%, *p = *0.016). Longitudinally, the image interpretation group demonstrated significant increase in the proportion of lipid‐lowering medication use from baseline to post‐enrollment (21.3% to 76.3%, *p* < 0.001) and from baseline to 3‐month follow‐up (21.3% to 72.2%, *p < *0.001), with a non‐significant decrease from post‐enrollment to 3‐month follow‐up (76.3% to 72.2%, *p = *0.46). The routine care group also showed significant increase from baseline to post‐enrollment (12.4% to 67.5%, *p* < 0.001) and from baseline to 3‐month follow‐up (12.4% to 59.2%, *p* < 0.001), with a non‐significant decrease from post‐enrollment to 3‐month follow‐up (67.5% to 59.2%, *p = *0.14).

## 4 Discussion

The findings of this study indicate that when GPs consult elderly patients with elevated ASCVD risk in primary care settings, incorporating imaging interpretation of carotid ultrasound results into physician–patient communication can better improve patients’ lipid management compared to routine care. At the 3‐month follow‐up, LDL, TC, TG, and HDL levels all decreased under the two care models, but the reduction was more pronounced in the image interpretation group. Notably, for LDL—the primary target of lipid management—both the LDL target attainment rate and lipid‐lowering medication adherence were significantly higher in the image interpretation group. The greater compliance to lipid‐lowering therapy in the image interpretation group aligns with a well‐documented dynamic in cardiovascular care—where tangible clinical evidence of disease drives adherence (Gargiulo et al. 2023; 2024).

Aging of the population has now become a fundamental condition in China and will remain so for a long time. A recent discussion published in the *British Medical Journal* pointed out that with the advent of aging, age‐related non‐communicable diseases will significantly increase the social and economic burden (Lv et al. 2024). Cardiovascular diseases are common non‐communicable diseases among the elderly and are also the main cause of death and disability, among which ASCVD is a major component. Guidelines recommend assessing the risk of ASCVD in primary prevention and controlling risk factors based on the risk to better prevent ASCVD (Li et al. 2023; Arnett et al. 2019). The Chinese 10‐year ASCVD Risk Assessment Flowchart classifies risks into low risk < 5%, intermediate risk 5%–9%, and high risk ≥ 10%. The corresponding LDL targets are set as < 3.4 mmol/L for low risk, < 2.6 mmol/L for intermediate and high risks, and < 1.8 mmol/L for special populations such as patients with diabetes over 40 years old. However, the results of the China Patient‐centered Evaluative Assessment of Cardiac Events Million Persons Project (China‐PEACE MPP) show that the LDL target attainment rate among high‐risk populations is only 46.8%, and nearly half of the high‐risk patients do not take lipid‐lowering medication (Bi et al. 2022). The population enrolled in this study was mainly elderly people at high risk of ASCVD, with the prevalence of hypertension, diabetes, and dyslipidemia all exceeding 50%. A large cross‐sectional survey based on China‐PEACE MPP shows that the prevalence of hypertension among high‐risk ASCVD populations is 96.2%, the prevalence of diabetes is 51.6%, and the prevalence of dyslipidemia is 54.9%, which is similar to the results of this study (Lu et al. 2019). The LDL target attainment rate of the population enrolled in this study at baseline was lower than that of the China‐PEACE MPP project. Reasons might be related to the low lipid‐lowering medication taking rate of the two groups at baseline, which indirectly reflects the insufficient practice of GPs in lipid management guidelines. LDL control in primary prevention can reduce the risk of ASCVD (Silverman et al. 2016).

The assessment of ASCVD risk not only serves to guide lipid management but is also frequently used as a tool for physician–patient communication to improve treatment adherence. However, a systematic review has indicated that there is currently no relevant evidence showing that risk assessment–based management in primary ASCVD prevention can reduce the incidence or mortality of cardiovascular diseases, and its improvement on risk factors is also limited (Collins et al. 2017). The reason may be related to the abstract nature of risk assessment results, which fail to promote patients to actively change their behaviors. To address this, researchers have proposed supplementing verbal explanations with visual stimuli, that is, incorporating the interpretation of imaging examination results into physician–patient communication.

Multiple previous clinical studies have explored integrating image information of atherosclerotic lesions into the current ASCVD prevention and treatment model. (Näslund et al. 2019) conducted an open‐label randomized controlled trial in Sweden, enrolling patients aged 40 and above with elevated ASCVD risk. The intervention group received carotid ultrasound examination in addition to routine treatment, and the image information was communicated to patients, while the control group only received routine treatment without providing image information. After 1 year of follow‐up, the Framingham Risk Score (FRS) of the control group was significantly higher than that of the intervention group (*β* = 1.07, 95% CI 0.11, 2.03), and the FRS within the intervention group was significantly lower than the baseline (*β* = −0.58, 95% CI −0.86, −0.3). Secondary outcome analysis found that the intervention could significantly reduce TC and LDL levels, but had no significant impact on HDL, systolic blood pressure, waist circumference, etc. Among them, the intervention effect on LDL was more significant in the population taking antihypertensive drugs. Rodondi et al. (2012) similarly using carotid ultrasound examination in the intervention group, did not find that the intervention could increase the smoking cessation rate, reduce FRS, or improve cardiovascular disease‐related risk factors after 1 year of follow‐up, which was considered possibly related to the fact that this study only enrolled smokers and used the smoking cessation rate as the primary outcome indicator. In our study, GPs interpreted imaging results and guided lipid management for patients. After 3 months of follow‐up, compared with patients in the control group receiving routine treatment, patients in the image interpretation group had a greater reduction in LDL, with a *β* coefficient of −0.38 (95% CI −0.71, −0.04), but there were no significant between‐group differences in changes in TC, HDL, and TG, which was similar to the results of Näslund et al. (2019). We also found that in patients with hypertension, the reduction in LDL in the image interpretation group was greater. At the same time, we found that image interpretation had a better effect on lipid management in patients with obesity, non‐smoking patients, and patients who did not take lipid‐lowering medications at baseline, suggesting that this intervention may be more effective in these types of patients. In the image interpretation group, although the results of carotid ultrasound examination were communicated and explained to all patients, whether carotid plaque was detected had an impact on the effect of lipid management. Patients with detected carotid plaque had greater reductions in LDL and TC, and a greater increase in HDL after 3 months of follow‐up. Although there was no difference in the impact on TG between the groups when comparing the 3‐month and baseline time points, the overall results indicated that the information of carotid plaque detected might have a greater impact on patients, prompting them to pay more attention to lipid management. From the perspective of GPs, understanding the carotid ultrasound examination results of patients also affects the treatment plans they provide to patients. From the changes in the use of lipid‐lowering medication by patients in the two groups at the three time points, it can be seen that GPs followed the recommendations of lipid management guidelines for populations with elevated ASCVD risk. Although the lipid‐lowering medication rate before the visit was low, the medication rate significantly increased after the enrollment. Among them, some patients still did not take medications due to concerns about side effects, but the treatment adherence of treated patients was significantly improved. From post‐enrollment to 3 months, the decrease in lipid‐lowering medication rate was not statistically significant, a finding consistent with the results of a post‐hoc analysis based on clinical trial data (Sjolander et al. 2021). Hong et al. (2014) also found that positive carotid plaque screening would prompt doctors to strengthen lipid management in hypertensive patients in primary prevention, while Johnson et al. (2011) found that carotid plaque screening could only change doctors’ behaviors, with insufficient impact on patients’ behaviors, possibly related to the fact that this study only followed up the enrolled population for 1 month and the outcome assessment was collected through questionnaires. In addition to using ultrasound examinations, some researchers have used CTA to assess coronary atherosclerosis as an intervention, and the results have shown that it can also achieve good results in primary prevention of ASCVD (Venkataraman et al. 2021; O'Malley et al. 2003; Rozanski et al. 2011).

### 4.1 Limitations

When interpreting the results of this study, the following limitations should be noted. First, the current study cannot fully disentangle the independent effects of carotid ultrasound image interpretation from the health counseling provided, which may have contributed to the observed improvements in lipid management. Second, this study lacks long‐term follow‐up data. Due to the short follow‐up period of only 3 months, it was unable to assess the impact of image interpretation on the incidence and mortality of ASCVD. Concurrently, the study did not evaluate the effect of image interpretation on patients’ lifestyles. The greater reduction in LDL observed in the image interpretation group at 3‐month follow‐up compared to baseline might also stem from patients’ active lifestyle modifications, such as a healthy diet and moderate exercise. Third, this was an observational study with imbalanced baseline characteristics between the two groups. Some patients in the routine care group received routine carotid ultrasound examination, and others might have previously undergone such examinations or known their carotid plaque status, which could introduce bias into the results. To address this, we adjusted for confounding factors in the GEE model, calculated propensity scores using PSM and incorporated them as covariates into the GEE model, and also included the factor of routine ultrasound exposure in the routine care group as a covariate. Although the results were consistent across these adjustments, we cannot exclude the influence of other unmeasured confounding factors (e.g., patients’ educational attainment). While unmeasured factors like health literacy may play a role, the consistency of effects across subgroups with varying baseline engagement suggests the intervention itself drives improvements. Randomized controlled trials are needed in the future to validate these findings. Fourth, the sample size of this study only met the requirements for the primary hypothesis test, so the results of subgroup analyses need to be validated in larger samples in the future. Fifth, although pooled analyses of randomized controlled trials indicate a low incidence of side effects from statins (Adhyaru and Jacobson 2018), when evaluating changes in the proportion of lipid‐lowering medication use across the three time points, we cannot exclude the potential impact of side effects on the results, which requires further evaluation in future studies. Finally, our findings may be more generalizable to Chinese primary care than foreign studies, as they reflect local GPs training and patient‐clinician dynamics. However, variability in GPs POCUS adoption across regions could limit broad application, highlighting the need for scalable training programs.

## 5 Conclusions

This study indicated that adding image interpretation of carotid ultrasound results to routine care by GPs significantly improves lipid management in elderly patients with elevated ASCVD risk.

## Author Contributions

**Xiaochuan Liu**: conceptualization, data curation, formal analysis, writing – original draft, writing – review and editing. **Sichen Yao**: data curation, formal analysis, writing – review and editing. **Hua Yang**: writing – review and editing, funding acquisition. **Zhigang Pan**: conceptualization, supervision, writing – review and editing. All the authors had read and approved the final manuscript.

## Conflicts of Interest

The authors declare no conflicts of interest.

## Supporting information




**Supplementary Information**: brb370956‐sup‐0001‐SuppMat.docx

## Data Availability

Original data of the study are available from the corresponding author upon reasonable request.
