# Supplementary material for: Impact of Image Interpretation of Carotid Ultrasound Findings on Lipid Management in Patients With Elevated ASCVD Risk
Source: Brain Behav. 2025 Nov 26;15(12):e70956. doi: 10.1002/brb3.70956 (PMC12650798; doi:10.1002/brb3.70956)
Supplement: Supplementary file 1 — Supplementary Information: brb370956‐sup‐0001‐SuppMat.docx [file BRB3-15-e70956-s001.docx]

**Supplementary material**

Carotid screening protocol

A standardized scanning protocol was established by ultrasound specialists. The specific procedure was as follows: (1) Patient lay supine on the examination bed with the head slightly tilted backward to expose the neck. Bilateral carotid arteries were scanned sequentially from right to left. (2) Using a linear ultrasound transducer, the common carotid artery (proximal, mid, and distal segments), carotid bifurcation, internal carotid artery, and external carotid artery were scanned from the cervical root. Transverse view scanning was performed first to observe intimal thickness changes and plaque/stenosis in the lumen, followed by longitudinal view scanning for intimal thickness and plaque measurements. The Intima-Media Thickness (IMT) of the carotid artery was measured 1.0-1.5 cm distal to the carotid bifurcation. (3) The built-in video recording function of the intelligent POCUS was activated throughout the entire scanning process. All recordings were uploaded to a cloud platform and assigned unique identifiers.

Inclusion and exclusion criteria for patients

Patients included in this study should meet the following standards: Elderly individuals aged ≥60 years; Elevated risk of ASCVD based on the ASCVD Risk Assessment Flowchart [11]; Agreed to participant. Patients meet these standards would be excluded: Pre-existing confirmed carotid plaque; Prior ASCVD; Cognitive impairments precluding study cooperation; Conditions limiting mobility; Significant carotid stenosis identified by POCUS; History of cervical vascular trauma or surgery; Refusal to participate.

Definition of carotid plaque

The definition of carotid plaque in this study adheres to established clinical consensuses. For reference, a normal carotid artery exhibits an intima-media thickness (IMT) of <1.0 mm. An IMT exceeding 1.0 mm is indicative of intimal thickening, whereas an IMT ≥1.5 mm confirms plaque presence. Alternatively, intimal thickening with a protrusion exceeding 50% of the adjacent area also indicates plaque formation.

Details of the intervention in Image Interpretation Group (active group)

The intervention was a structured, GPs-led process integrating carotid ultrasound screening, visual image interpretation, and targeted lipid management guidance, conducted during an initial consultation (with follow-up at 3 months). The key steps were as follows:

(1) Carotid ultrasound screening: GPs (trained in using intelligent point-of-care ultrasound [POCUS] per our prior study) performed bilateral carotid artery scans. The intelligent POCUS system automatically identified and highlighted carotid plaques (if present) with on-screen markers, ensuring standardized visualization.

(2) Visual image interpretation: After screening, GPs reviewed the ultrasound images with patients in real time. Using the visualized plaques (or absence thereof) as a teaching tool, GPs explained:

The meaning of findings (e.g., “This bright area on the image is a plaque, which is a buildup of fat and cholesterol in your artery wall”); The link between plaques and ASCVD risk (e.g., “Plaques can narrow arteries over time, increasing the risk of heart attack or stroke, which is why managing your cholesterol is critical”); How lipid control (via medications and lifestyle) can stabilize or slow plaque progression.

(3) Comprehensive lipid management education: In addition to image-based discussion, GPs provided personalized guidance aligned with the Chinese lipid management guidelines. including: Tailored LDL target recommendations; Explanation of lipid-lowering medications; Lifestyle advice with examples relevant to elderly patients.

Documentation and follow-up plan: The ultrasound findings and management plan were documented in the patient’s medical record. Patients were scheduled for a 3-month follow-up to reassess lipid levels, medication adherence, and adjust management as needed.

Sample size consideration

This observational cohort study aims to explore the impact of image interpretation based on carotid ultrasound examination by GPs on lipid management, with the primary outcome being the between-group difference in LDL changes from baseline to 3-month follow-up. Previous randomized controlled trial of similar interventions showed that the intervention group had a more significant LDL reduction compared to the control group after one-year follow-up, with a between-group difference of approximately 0.3 mmol/L. Given that this study enrolls elderly patients with elevated ASCVD risk and has a relatively short follow-up period, the lipid management effect in the image interpretation group is expected to be better. Thus, the expected between-group difference in LDL changes was increased by 20% to 0.36 mmol/L. With a power of 80%, a type I error rate of 0.05, an LDL autocorrelation coefficient of 0.3 based on our small-sample pilot study, and a standard deviation of 0.9, the PASS 15.0 software calculation showed that at least 99 participants were required per group. Considering a 10% dropout rate, at least 110 participants per group were needed, resulting in a total sample size of at least 220.

Sensitivity analysis

To test the reliability of our results, we first applied propensity score matching (PSM), a commonly used method in clinical research to balance between-group differences. Using study group as the dependent variable and baseline characteristics as independent variables, we employed nearest neighbor matching with a caliper value of 0.03. A logistic regression model was constructed to calculate propensity scores, which were then included as covariates in the GEE model to examine whether the study findings changed. Secondly, a small subset of patients in the routine care group underwent routinely paid carotid ultrasound examination, and GPs conducted physician-patient communication based on the examination reports. We included this as a covariate in the GEE model to test its impact on the study outcomes.

Table 1 Impact of carotid plaque detection on outcomes in image interpretation group

|  |  | Without Carotid Plaque | With Carotid Plaque |  |  |  |  |
| --- | --- | --- | --- | --- | --- | --- | --- |
|  |  | 92 | 77 | P | interaction P | β | 95% CI |
| Primary outcome | |  |  |  |  |  |  |
| LDL | Baseline | 2.93 (2.09, 3.1) | 2.93 (2.1, 3.98) | 0.18 | ＜0.001 | -0.87 | (-1.33,-0.42) |
|  | 3-month follow up | 2.67 (1.29, 3.31) | 1.3 (1.02, 2.89) | ＜0.001 |  |  |  |
| Secondary outcomes | |  |  |  |  |  |  |
| TC | Baseline | 4.79 (3.49, 5.66) | 4.79 (4.04, 5.92) | 0.26 | 0.029 | -0.59 | (-1.12,-0.06) |
|  | 3-month follow up | 4.11 (3.15, 5.05) | 3.47 (2.64, 4.65) | 0.034 |  |  |  |
| TG | Baseline | 1.84 (0.81, 3.06) | 2.93 (2.01, 3.67) | ＜0.001 | 0.53 | -0.37 | (-1.54,0.79) |
|  | 3-month follow up | 1.76 (1.46, 2.11) | 1.76 (1.46, 1.97) | 0.77 |  |  |  |
| HDL | Baseline | 1.27 (0.93, 1.91) | 1.28 (0.95, 1.62) | 0.99 | 0.001 | 0.63 | (0.26,1.0) |
|  | 3-month follow up | 1.18 (0.97, 1.54) | 1.74 (1.18, 2.3) | ＜0.001 |  |  |  |

Note: LDL: low-density lipoprotein; TC: total cholesterol; TG: triglyceride; HDL: high-density lipoprotein. Lipid indices are in mmol/L. CI: confidence interval. The P value represents the statistic for between-group comparison of each index at two time points. The interaction P value is the statistic for the interaction term constructed by group (routine care group/image interpretation group) and time (baseline/3-month follow-up). Model 1 is the original model, and Model 2 is adjusted for confounders, including age, hypertension, diabetes, and baseline lipid-lowering medication use.

Table 2 Subgroup analysis of the impact of image interpretation on LDL

|  |  | Routine care group | Image interpretation group | P | interaction P | β | 95% CI |
| --- | --- | --- | --- | --- | --- | --- | --- |
| Subgroup |  | N | N |  |  |  |  |
| Gender | Male | 64 | 64 |  | 0.131 | -0.42 | (-0.96,0.12) |
|  | Baseline | 2.93 (2.1, 3.1) | 2.88 (2.07, 3.28) | 0.21 |  |  |  |
|  | 3-month follow up | 3.17 (1.43, 3.93) | 1.91 (1.17, 3.08) | 0.006 |  |  |  |
|  | Female | 105 | 105 |  | 0.107 | -0.35 | (-0.77,0.07) |
|  | Baseline | 2.93 (2.1, 3.45) | 2.93 (2.1, 3.45) | 0.84 |  |  |  |
|  | 3-month follow up | 2.68 (1.35, 3.67) | 1.71 (1.18, 3.19) | 0.018 |  |  |  |
| Hypertension | Yes | 133 | 112 |  | **0.03** | -0.43 | (-0.81,-0.04) |
|  | Baseline | 2.93 (2.1, 3.45) | 2.93 (2.09, 3.45) | 0.55 |  |  |  |
|  | 3-month follow up | 2.88 (1.35, 3.69) | 1.53 (1.17, 3.1) | 0.001 |  |  |  |
|  | No | 36 | 57 |  | 0.264 | -0.39 | (-1.08,0.29) |
|  | Baseline | 2.93 (2.1, 3.1) | 2.93 (2.09, 3.1) | 0.98 |  |  |  |
|  | 3-month follow up | 3.03 (1.49, 3.82) | 2.61 (1.24, 3.25) | 0.11 |  |  |  |
| Dyslipidemia | Yes | 106 | 108 |  | 0.159 | -0.31 | (-0.75,0.12) |
|  | Baseline | 2.93 (2.1, 3.45) | 2.93 (2.09, 3.72) | 0.84 |  |  |  |
|  | 3-month follow up | 2.89 (1.44, 3.79) | 1.91 (1.18, 3.23) | 0.046 |  |  |  |
|  | No | 63 | 61 |  | 0.061 | -0.49 | (-0.99,0.02) |
|  | Baseline | 2.93 (2.1, 3.1) | 2.93 (2.09, 3.1) | 0.22 |  |  |  |
|  | 3-month follow up | 2.9 (1.28, 3.76) | 1.46 (1.16, 2.99) | 0.003 |  |  |  |
| Diabetes | Yes | 110 | 93 |  | 0.168 | -0.3 | (-0.74,0.13) |
|  | Baseline | 2.93 (2.1, 3.1) | 2.88 (1.97, 3.1) | 0.17 |  |  |  |
|  | 3-month follow up | 2.83 (1.28, 3.68) | 1.48 (1.11, 3.01) | 0.003 |  |  |  |
|  | No | 59 | 76 |  | 0.074 | -0.48 | (-1.01,0.05) |
|  | Baseline | 2.93 (2.1, 4.11) | 2.93 (2.1, 3.72) | 0.74 |  |  |  |
|  | 3-month follow up | 2.92 (1.6, 3.99) | 1.91 (1.24, 3.37) | 0.034 |  |  |  |
| Obesity | Yes | 25 | 29 |  | **0.007** | -1.1 | (-1.91,-0.30) |
|  | Baseline | 3.05 (2.54, 4.12) | 3.01 (2.62, 3.45) | 0.92 |  |  |  |
|  | 3-month follow up | 3.33 (2.79, 4.12) | 1.39 (1.18, 3.05) | 0.001 |  |  |  |
|  | No | 144 | 140 |  | 0.206 | -0.23 | (-0.6,0.13) |
|  | Baseline | 2.93 (2.1, 3.1) | 2.93 (2.09, 3.1) | 0.47 |  |  |  |
|  | 3-month follow up | 2.75 (1.27, 3.68) | 1.81 (1.18, 3.17) | 0.018 |  |  |  |
| Smoking history | Never smoked | 131 | 135 |  | **0.026** | -0.42 | (-0.8,-0.05) |
|  | Baseline | 2.93 (2.1, 3.45) | 2.93 (2.1, 3.45) | 0.59 |  |  |  |
|  | 3-month follow up | 2.77 (1.29, 3.67) | 1.71 (1.16, 3.16) | 0.006 |  |  |  |
|  | Previously smoked, currently quit | 21 | 21 |  | 0.37 | -0.38 | (-1.22,0.46) |
|  | Baseline | 2.93 (2.1, 3.1) | 2.1 (1.82, 2.93) | 0.062 |  |  |  |
|  | 3-month follow up | 3.14 (1.52, 3.65) | 1.41 (1.18, 2.86) | 0.051 |  |  |  |
|  | Currently smoking | 17 | 13 |  | 0.739 | 0.21 | (-1.01,1.43) |
|  | Baseline | 2.93 (2.62, 4.55) | 2.54 (1.97, 4.11) | 0.21 |  |  |  |
|  | 3-month follow up | 3.65 (2.66, 3.99) | 2.96 (1.96, 3.44) | 0.38 |  |  |  |
| Lipid-lowering medication use at baseline | Yes | 21 | 36 |  | 0.051 | 0.88 | (0,1.77) |
|  | Baseline | 2.54 (1.97, 4.11) | 2.71 (1.9, 3.01) | 0.87 |  |  |  |
|  | 3-month follow up | 1.23 (0.98, 1.79) | 1.92 (1.2, 3.18) | 0.04 |  |  |  |
|  | No | 148 | 133 |  | **0.001** | -0.6 | (-0.95,-0.25) |
|  | Baseline | 2.93 (2.1, 3.1) | 2.93 (2.1, 3.45) | 0.78 |  |  |  |
|  | 3-month follow up | 3.12 (1.49, 3.79) | 1.7 (1.16, 3.1) | <0.001 |  |  |  |

Bold fonts indicate statistically significant interaction P values. LDL is measured in mmol/L. CI: confidence interval. The P value represents the statistic for between-group comparison of LDL at two time points, and the interaction P value is the statistic for the interaction term constructed by group (routine care group/image interpretation group) and time (baseline/3-month follow-up).

Table 3 Sensitivity analysis of the impact of image interpretation on Lipid management

|  | Model 1 |  |  | Model 2 |  |  |
| --- | --- | --- | --- | --- | --- | --- |
|  | interaction P | β | 95% CI | interaction P | β | 95% CI |
| Primary outcome |  |  |  |  |  |  |
| LDL | 0.027 | -0.38 | (-0.71,-0.04) | 0.025 | -0.38 | (-0.7,-0.05) |
| Secondary outcomes |  |  |  |  |  |  |
| TC | 0.514 | 0.12 | (-0.25,0.5) | 0.515 | 0.12 | (-0.25,0.5) |
| TG | 0.964 | -0.02 | (-0.71,0.68) | 0.964 | -0.02 | (-0.71,0.68) |
| HDL | 0.686 | 0.05 | (-0.19,0.3) | 0.686 | 0.05 | (-0.2,0.3) |

Note: CI: confidence interval; Model 1 was adjusted for propensity score; Model 2 was adjusted for patients who underwent routine ultrasound examination in the routine care group; the interaction P value was the statistic of the interaction term constructed by group (routine care group/image interpretation group) and time (baseline/3-month follow-up).


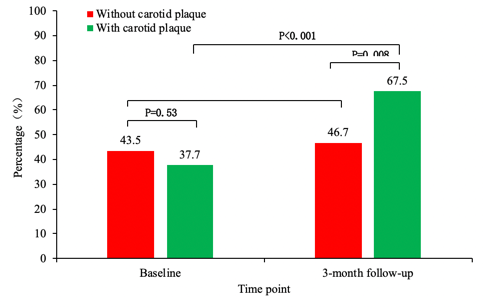


Figure 1 Impact of carotid plaque detection on LDL target attainment rate

References

Touboul PJ, Hennerici MG, Meairs S, Adams H, Amarenco P, Bornstein N, et al. Mannheim carotid intima-media thickness and plaque consensus (2004-2006-2011). An update on behalf of the advisory board of the 3rd, 4th and 5th watching the risk symposia, at the 13th, 15th and 20th European Stroke Conferences, Mannheim, Germany, 2004, Brussels, Belgium, 2006, and Hamburg, Germany, 2011. Cerebrovasc Dis. 2012;34(4):290-6. Epub 2012/11/07. doi: 10.1159/000343145. PubMed PMID: 23128470; PubMed Central PMCID: PMCPMC3760791.

Li JJ, Zhao SP, Zhao D, Lu GP, Peng DQ, Liu J, et al. 2023 Chinese guideline for lipid management. Front Pharmacol. 2023;14:1190934. Epub 2023/09/15. doi: 10.3389/fphar.2023.1190934.

Naslund U, Ng N, Lundgren A, Fharm E, Gronlund C, Johansson H, et al. Visualization of asymptomatic atherosclerotic disease for optimum cardiovascular prevention (VIPVIZA): a pragmatic, open-label, randomised controlled trial. Lancet. 2019;393(10167):133-42. Epub 2018/12/14. doi: 10.1016/S0140-6736(18)32818-6.

Austin PC. An Introduction to Propensity Score Methods for Reducing the Effects of Confounding in Observational Studies. Multivariate Behav Res. 2011;46(3):399-424. Epub 2011/08/06. doi: 10.1080/00273171.2011.568786.
